# Decision-Making Processes in Surrogates of Cancer Patients in a Taiwan Intensive Care Unit

**DOI:** 10.3390/ijerph17124443

**Published:** 2020-06-20

**Authors:** Wan-Na Sun, Hsin-Tien Hsu, Nai-Ying Ko, Yu-Tung Huang

**Affiliations:** 1 Department of Nursing, National Cheng Kung University Hospital, College of Medicine, National Cheng Kung University, Tainan 704, Taiwan; nymph198722@gmail.com (W.-N.S.); hivedu.ncku@gmail.com (N.-Y.K.); 2 School of Nursing, College of Nursing, Kaohsiung Medical University, Kaohsiung 807, Taiwan; 3 Department of Medical Research, Kaohsiung Medical University Hospital, Kaohsiung Medical University, Kaohsiung 807, Taiwan; 4 Center for Big Data Analytic and Statistics, Chang Gung Memorial Hospital, Taoyuan 333, Taiwan; anton.huang@gmail.com

**Keywords:** decision-making process, surrogates, cancer patients, intensive care unit (ICU)

## Abstract

*Background*: Few studies in Asian countries have explored the emotional entanglements and conflicts that surrogates often experience during the medical decision-making process. This study was to explore decision-making processes in surrogates of cancer patients in a Taiwan intensive care unit (ICU). This qualitative study surveyed a purposive sample of surrogates (*n* = 8; average age, 48 years) of cancer patients in the ICU of a medical center in Taiwan. A phenomenological methodology was used, and a purposive sample of surrogates of cancer patients were recruited and interviewed during the first three days of the ICU stay. *Results*: Based on the interview results, four themes were generalized through text progression: (1) Use love to resist: internal angst. This theme was related to the reflexive self -blame, the feelings of inner conflict, and the reluctance to make healthcare decisions, which surrogates experienced when they perceived suffering by the patient. (2) Allow an angel to spread love among us: memories and emotional entanglements. Memories of the patient caused the surrogate to experience emotional entanglements ranging from happiness to sadness and from cheerfulness to anger. (3) Dilemmas of love: anxiety about ICU visitor restrictions. The confined space and restricted visiting hours of the ICU limited the ability of surrogates to provide emotional support and to share their emotions with the patient. (4) Suffocating love: entanglement in decision-making. Emotional entanglements among family members with different opinions on medical care and their struggles to influence decision-making often prevented surrogates from thinking logically. Conclusions: Expression of emotions by ICU surrogates is often restrained and implicit, particularly in Asian populations. These results can help health professionals understand the psychological shock and inner conflict experienced by surrogates and provide a useful reference for improving their communications with surrogates.

## 1. Introduction

Each year, 8.2 million people worldwide die of cancer; most (70%) of these cancer deaths occur in Asia [1]. Cancer patients often require transfer to intensive care units (ICUs) after they undergo invasive procedures such as cardiopulmonary resuscitation and tracheotomy or when they suffer comorbidities such as acute respiratory failure, infection induced by disease progression or treatment, cardiovascular disorders, and neurological disorders [2,3]. The patient or the surrogate (if the patient lacks clear consciousness) may inform the medical team that palliative care is acceptable. After signing a statement in accordance with the Hospice Palliative Care Act, the patient is transferred to a hospice ward or a palliative care ward according to the condition of the patient. Policies for ICU visits differ by country and by institution. In Taiwan, for example, most ICUs limit visits to 30 min two times a day and by no more than two visitors at a time. The rationale is to protect the privacy of the patients, control infection, and minimize interference with treatment. In comparison, visitor policies are relatively more flexible in the United States but relatively stricter in the UK and elsewhere in Western Europe [4].

For cancer patients who lack capacity to make decisions about their medical care while in ICU, healthcare decisions are often made by a surrogate or relatives. Nearly 95% of cancer patients cannot make their own medical decisions at the time of ICU admission and must rely on a surrogate [5]. In Taiwan, a patient under clear consciousness can sign a letter of appointment for a pre-determined surrogate. A surrogate is defined as a family member, friend, or other person who communicates the preferences of the patient regarding medical treatment and makes major medical decisions for the patient, such as consenting to life-sustaining treatments, end-of-life care, etc. Decision-making abilities include the abilities to understand and clearly express ideas, to make decisions about treatment, and to evaluate and debate the merits of available treatment options [6]. In Taiwan, a surrogate must be aged 20 years or older and have behavioral capacity to make healthcare-related decisions on behalf of the patient. Legally, surrogates appointed by medical institutions have the right to make medical decisions for the patient, even if family members disagree. If a patient has not signed a letter of appointment for a pre-determined surrogate, healthcare-related decisions can be made by a legal relative [7,8,9].

An ICU admission is a crisis situation for both the patient and the surrogate. For example, 14–81% of the family members of ICU patients have symptoms of posttraumatic stress disorder after they participate in making healthcare decisions for the patient [5]. In a study of stress experienced by family members of ICU patients treated at a Taiwan medical center, Li (2017) found that stress in family members peaked within 48 h after the ICU transfer [10]. Contributing factors include the unfamiliar environment of the ICU, fatigue, negative emotions (e.g., hopelessness, anxiety, and fear), and limited understanding of the knowledge aspects of the treatment. This stress can cause strain in family relationships or friendships, disputes over medical decisions, and poor healthcare-related decisions by surrogates. Additionally, some populations of patients (e.g., elders living alone, unmarried people, and sexual and gender minorities) may not have a strong personal affinity with their legal surrogates. Surrogates for these populations may endure inner conflict during decision-making and during efforts to reach a family consensus. The combination of a complex medical condition and a complex family relationship further increases the possibility of decision-making conflicts.

The decision-making process in ICU surrogates may differ by culture, and culture directly affects individual behaviors. For example, social harmony is the essence of Confucian theories of social interaction in Chinese culture. Chinese society is relational and collective. The social interactions and behaviors of all members of society are guided by Confucian social norms, which make social interaction more complex compared to that in western culture [11,12]. All relationships in a Confucian society can be classified into five types: superior and subordinate, father and son, husband and wife, elder brother and younger, and friends. Each person in a social relationship has a personal responsibility to maintain the relationship. Social goals also represent different types and levels of love. According to the theory of love proposed by the social psychologist Zick Rubin in 1970, love is the deepest and most meaningful of sentiments. Love is also a multifaceted attitude that a person has about another [13]. Since love is an abstract concept, however, the definition of love depends on the perspective of the individual. That is, the definition of love differs because individual feelings, behaviors, and attitudes differ [14]. Another theory of love is the Sternberg “triangular” theory. According to Sternberg, love has three components: intimacy, passion, and decision/commitment. Each type of love can occur in an interactive and dynamic intimate relationship. In western culture, contact between two people, including physical contact and even eye contact, indicates a strong relationship (deep love) [13,14]. Different types of love are also recognized in Chinese culture. With the exception of friendships, all five of the Confucian relationships are vertical relationships, e.g., “respect” versus “humble” and “up” versus “down”. Therefore, in Chinese culture, emotions are expressed in a roundabout and restrained manner in all relationships except friendships.

Because of their love, ICU surrogates worry about losing patients. The roles and relationships of family members also change when they must make healthcare decisions that could determine whether or not the patient survives. In this situation, families experience anxiety and other negative emotions [15]. Research indicates that 20–46% of cancer patients meet the clinical criteria for depression and worry, and up to 75% of spouses experience negative emotions [10]. Chinese populations often have a sense of responsibility and obligation to the family or social group. That is, family and social relationships are prioritized, unlike Western culture, in which the independence of the individual and the freedom to make choices are valued and prioritized [11,12]. Therefore, family and social relationships and attempts to integrate with the family or a social group can cause psychological distress. 

Cultural attitudes about medical decision-making may be different. For example, Dionne-Odom et al. (2015) investigated decision-making experiences in 19 primary surrogate decision-makers of patients in critical care units in the rural Northeastern United States. In these surrogates, decision-making was impacted by gist impressions, distressing emotions, and moral intuitions [16]. In another study in the United States, Moss et al. (2019) interviewed seven surrogates (four Caucasian and three African American) and deduced three major themes of the decision-making process: “communication as key in decision making”, “impact of past experiences”, and “difficulties and coping” [17]. Asians traditionally have low completion rates for advance care planning (ACP) and tend to rely on the family model of medical decision-making. Su et al. (2014) investigated the decision-making experience in 69 medical care surrogates (29% African American, 26% White, 26% Asian/Pacific Islander, and 19% Latino) and identified six subthemes, including two Communication subthemes (unspoken expectations and discussion of death as taboo), two Emotion subthemes (emotional stress and feelings of loneliness), and two Conflict subthemes (family conflict and potential solutions for preventing conflict) [18]. These studies indicate that cultural attitudes about medical decision-making and filial expectations may cause some surrogates to experience stress and family conflict.

Until now, most studies of surrogate decision-making for cancer patients in ICUs have been performed in western countries. Seldom studies have considered how surrogate decision-making is affected by cultural aspects unique to Asian populations such as the reluctance to discuss the impending death of a family member, which is considered taboo in Chinese culture. Even surrogates who recognize that discussion with other family members would improve the decision-making process for loved ones with cancer may avoid discussing these matters until a poor outcome occurs, e.g., coma or sudden death. Although ICU medical teams routinely assist surrogates in making medical decisions, the decision-making process in surrogates of cancer patients in ICU are not well understood. One study explored the quality of care delivered to ICU patients at the end stage showing end-of-life signs and symptoms. The authors reported that, although ICU personnel can determine whether or not death is inevitable, the ICU environment limits opportunities for surrogates to communicate with healthcare professionals. Lack of advance care planning (ACP) or lack of other documentation that expresses the will of the patient (e.g., do-not-resuscitate order) places the decision-making burden on the surrogate. Surrogates have insufficient time to bear the decision related to patient’s bad outcome, leading to poor quality in the face of patient’s death for surrogates confusing [19].

In Taiwan, ACP has not yet matured. The Hospice Palliative Care Act can only be signed for patients with terminal cancer, and the Patient Right to Autonomy Act was only recently passed in 2019 [6]. However, ACP has limitations. First, most patients in critical care units in Taiwan are highly reliant on their surrogates. For patients with complex clinical conditions, accurately predicting the outcomes of a medical treatment is often difficult. The preferred treatments indicated by patients during the ACP process may later prove impractical or inapplicable. Additionally, patients may not anticipate various outcomes when they indicate their preference for active treatment. For example, during ACP, the active treatment that a patient selects for progression of cancer may not be appropriate if the patient also has pneumonia. Preferences for active treatment indicated in ACP may not be a good choice for the patient and may later present a dilemma for the surrogate. For patients who do not sign an ACP or who do not sign a “do-not-resuscitate“ order, family members, especially in Asia, may prefer that the patient continue to live for reasons such as their strong emotional bond with the patient, pending property issues, etc., even if it is not in the best interest of the patient. Although longevity is considered a blessing in Confucian society, family harmony is equally important in Asia. Regardless of their current relationship, all family members desire harmony and may make a decision inconsistent with the preferences of the patient. Differences in family relationship, filial piety, etc. result in a decision-making experience very different from that in western culture [20]. Studies of medical decision-making for acute care patients or for children have elucidated the highly complex and multifaceted process of surrogate decision-making. However, little is known about the experience, the feelings, and the needs of surrogates of cancer patients in ICUs in Asia. Therefore, the objective of this study was to explore the experience of surrogates of cancer patients in ICU during the medical decision-making process.

## 2. Methods 

### 2.1. Design, Setting, and Participants

According to Denzin and Lincoln (2015), six respondents are sufficient to achieve data saturation when in-depth interviews are used to collect data in a phenomenological study [21]. This qualitative study interviewed surrogates (*n* = 8) as part of a descriptive, comparative study (*N* = 115). The researchers performed face-to-face interviews of surrogates of adult cancer patients who were currently in ICU and lacked decision-making capacity. A phenomenological methodology was used to understand the experience of surrogates in the course of decision-making. The inclusion criteria were designation as surrogate authorized to make medical decisions for a cancer patient currently in ICU and age over 20 years old. Surrogates were excluded if they had any emotional or cognitive deficiency. The principles applied in the subject selection process were high variability and good narrative skills. Data collection continued until data saturation was reached, i.e., when data analysis revealed no new information. The study was performed in a medical center in southern Taiwan with 1200 hospital beds, including 130 internal medicine and surgical ICU beds. At this medical center, cancer patients comprise 15.4% of all patients admitted to the ICU annually.

### 2.2. Data Collection

Institutional review board approval was obtained before this study was performed (A-ER-105-231). Surrogates who met the enrollment criteria were contacted, informed consent was obtained, and interviews were arranged. Before the interviews, the researchers explained the objective and procedure of the study to each subject. The subjects were informed that a researcher would record each interview and take notes during the interview. Surrogates were interviewed in conference rooms near the ICU. Each interview lasted 40–60 min. The subjects were ensured that all interview data would be anonymous, and all subjects gave written informed consent to participate in the study. An experienced researcher asked four questions in semi-structured interviews: (1) How would you describe your experience of the healthcare decision-making process during this ICU stay? (2) How would you describe your relationship with the patient during this ICU stay? (3) How have your daily life activities changed during this ICU stay? (4) How have you coped with emotions and experiences during this ICU stay?

Interviews were performed during the first 3 days of the ICU stay. At the end of the interview, each patient was given a gift as a token of appreciation for participating in the study.

### 2.3. Data Analysis 

Content analysis of interview data was performed in the following steps. First, data were encoded to ensure confidentiality of the interviewee. Each interview was recorded, and the researchers transcribed the audio recording of each interview within 48 h. Additionally, the researchers carefully read the resulting transcripts and extracted significant statements related to new meanings that emerged after transcription. The common characteristics of meanings were then clustered, and subthemes were grouped as themes [22]. The strategies used in this process included peer review, in which the researchers individually coded and then collectively negotiated mutually acceptable statements and definitions. Consistency in developing the major themes and subthemes was further enhanced by inviting an independent researcher to identify themes by reading transcripts of two randomly selected interviews. Content analysis was performed simultaneously with data collection. Data collection ended when theoretical saturation was achieved, i.e., when no new themes emerged in further data analysis. Three experts, each with 15–20 years of experience in qualitative research, reviewed and discussed randomly selected interview transcripts and the results of qualitative analysis performed by the principal investigator (PI). Finally, the qualitative analysis results were verified by further interviews and by comparisons with medical records. 

### 2.4. Rigor

The reliability and validity of the qualitative study were evaluated in terms of credibility, conformability, dependability, and transferability, which are well established standards for assessing the rigor of qualitative research [23,24,25]. Credibility: The PI had approximately 9 years of clinical experience in ICU and was in constant contact with the surrogates throughout the study. For an improved understanding of the phenomena investigated in this study, the PI completed an 8-month clinical practicum in palliative care wards, oncology outpatient departments, and surgical ICUs at the research sites to observe surrogate decision-making processes in various situations. Conformability: Interview data were collected by audio recordings and stenography. After the interviews, the PI confirmed the accuracy of the collected data by listening to the recordings of the interviews and comparing them with the written transcripts. The PI then asked each interviewee to clarify any unclear or ambiguous content. Dependability: To ensure high dependability of the research results, the qualitative experts reviewed selected interview transcripts and compared their analysis results with those obtained by the researcher. Transferability: The PI took handwritten notes regarding transient details of the interview process to provide a reference for further studies by other researchers. The PI and the qualitative experts then agreed on a coding structure. In the case of a difference of opinion on coding, the PI and qualitative experts continued discussion of the coding until a consensus was reached.

## 3. Results

**Figure 1:**
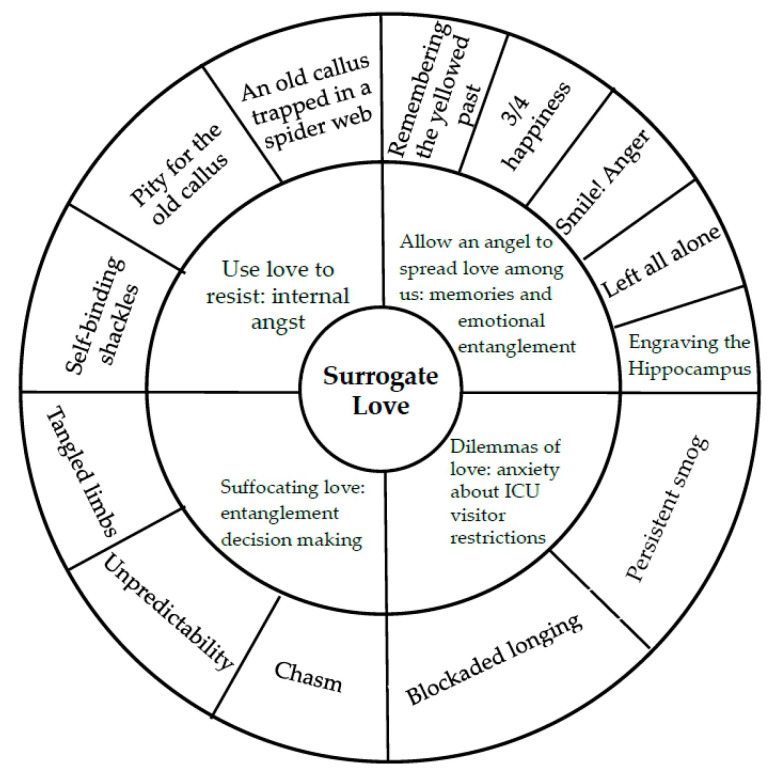
Themes and subthemes of surrogate decision-making process.

The subjects of this study were recruited during a six-month period from June to December 2017. Eight surrogates (average age, 48 years) were interviewed. Table 1 lists the basic information for the surrogates. Figure 1 shows the four themes, thirteen subthemes, and the meanings of each subtheme.

### 3.1. Use Love to Resist: Internal Angst

The internal angst theme relates to the reflexive self-blame, the inner conflict, and the reluctance to make decisions, which the surrogates experienced when they observed the patient suffering. Figure 1 shows the three subthemes of Use love to resist: internal angst.

#### 3.1.1. Self-Binding Shackles

When faced with the possible loss of a loved one, surrogates reflexively blamed themselves, as if hoping to reduce or compensate for the pain and suffering experienced by the patient. For example, Subject A stated that she usually traveled with her oldest daughter every summer. However, a sudden change in the condition of Subject A required a transfer to ICU for treatment. Therefore, Subject A and her daughter returned from their vacation in Japan on the day after they arrived. Subject A said, “I think to myself that, if I had not gone on vacation, none of this would have happened; I would not have been sent here….”

#### 3.1.2. Pity for the Old Callus 

Despite their short visiting hours with the patients, the surrogates could clearly discern the declining health and changing appearance of the patients as various tubes and other life support equipment and devices were attached to their bodies. The increasing use of medical equipment drew attention to the changing physical appearance of the patient and caused the surrogates to experience feelings of pity. For example, some patients required high doses of vasopressors to treat peripheral arterial occlusive disease. Subject G became concerned when she noticed that the patient had a spot on his hand after vasopressor treatment and said, “Yesterday, this spot was not this dark and not this big. I thought this hand was already as bad as it could get, but you can see how dark it is now. It is even worse than before.”

#### 3.1.3. An Old Callus Trapped in a Spider Web

When a patient is admitted to ICU, the surrogate may experience a stabbing heart pain caused by the perception that the patient is hanging on a cliff and is as fragile as an old callus. However, a surrogate must be willing to release the emotional attachment to a patient who is approaching death so that the patient can die peacefully with minimal pain and suffering. The decision to let go can cause immense stress for the surrogate. Subject B recalled, “I said in his ear that it was okay if he did not want to live. I told him I would be fine alone if he just followed the Buddha (cries).”

### 3.2. Allow an Angel to Spread Love Among Us: Memories and Emotional Entanglements

The surrogates in this study discussed their good memories of the patient. The semi-private nature of the ICU environment limited physical contact between the surrogate and the patient. The surrogate was similar to an “angel” who can comfort and accompany the patient. However, their emotional entanglements with the patient varied from happiness to sadness and from cheerfulness to anger. This study identified five subthemes (Figure 1).

#### 3.2.1. Remembering the Yellowed Past

While waiting outside the ICU, the surrogates often reflected on their past relationships with the patients. They contemplated the times they felt harmony as well as the times they had fights and arguments. Subject E said tearfully, “Whenever I go home at night to sleep, whenever I close my eyes, I can hear him calling me. I think about how we used to spend time together. Everyone envied us, you know…how am I supposed to fall asleep?”

Most of the surrogates had participated in important moments in the lives of the patients. Therefore, surrogates tended to associate the current suffering with unhappy experiences of the patient before hospitalization. Subject A described experience, “He knew that the drug therapy would be very difficult to endure, but he never lost his temper, and he always cooperated with the medical personnel until now. We always knew that, together, we could overcome depression or negative emotions.”

#### 3.2.2. 3/4 Happiness

If “1” represents a complete family with caring and mutually supportive family members, then “3/4” represents a family in which one member (1/4) is missing. When the patient is hospitalized, family members and the surrogate are busy traveling between the hospital and their disrupted home. Subject A said, “During this period (sigh), everyone has been running back and forth. I am thankful to have them now that patient is in ICU. Otherwise, I wouldn’t know what to do.” Subject E described a similar experience, “If we were to go home now, the thing we would fear most is the phone ringing. We would always be afraid that the hospital was calling.” Surrogates expressed fear of losing the patient and the willingness to do anything, no matter how difficult, to help the patient (1/4 of the family) get better and return home so that the family could be whole again. 

#### 3.2.3. Smile! Anger

For a patient whose body is wracked with the pain of a disease, emotions and moods can change. Surrogates hope for the best and continue smiling while caring for their loved ones, even when anger and conflicts arise. The surrogate considers the disease, pain, and low spirits of the patient and hopes for improvement in the condition of the patient. Subject H angrily said, “At home, I ask him what he wants to eat before I start cooking, and he does not respond! He does not like talking! Sometimes I get very angry serving him every day; I am also exhausted, but I am still willing to do anything for him (smiles).” Even when the patients tire and cry during the course of caring, the surrogates grudgingly tell themselves there is no other way and that caring for the patient is their responsibility. 

#### 3.2.4. Left all Alone

The formation of a traditional family starts with two individuals who know each other and who care for and love each other. They live together, rely on each other, and accompany each other. After the departure of a loved one, the surviving partner or family member must contemplate the prospect of continuing alone. Subject E lost his wife to recurrent breast cancer two years earlier. He and his son were the only remaining family members. Subject E said, “Two years ago my wife passed away, I have yet to walk out of that pain. Now my son is dying. There is no one left in the family.”

Family structures in Taiwan and throughout the world have rapidly changed in recent years. For example, the number of households with non-traditional family structures (e.g., double income, no kids (DINKs), singles, elders living alone, and gay couples) has increased. Changes in social welfare systems and legal systems may be too slow to accommodate these emerging populations. Current laws do not ensure comprehensive care for such populations on a daily basis. 

Surrogates are often under extreme stress, especially when they have no one to consult and no way to express their feelings. For example, Subject H, who had immigrated to Taiwan 20 years earlier, provided daily care for a patient. Subject H tearfully described the feelings she experienced when the patient was suddenly admitted to the ICU due to the disease. “Others had help, but I had no one to turn to. I felt so sad. Others had family members, but I was alone. Others had at least one or two people for support, but I was running in and out of ICU by myself. Do you know how desperate I felt?”

#### 3.2.5. Engraving the Hippocampus

In the current era of ubiquitous media and communications technology, family members may have difficulty remembering the date that a loved one was hospitalized. Surrogates, however, can often remember the date of every important disease-related event in the life history of the patient. Subject A, for example, could remember the date the patient was intubated and the days she waited for the patient to awake from sedation. These dates were engraved on her hippocampus. During the interview, Subject A said, “Yes, it has been a week since…was sedated last Wednesday; it was also a week last time.”

### 3.3. Dilemmas of Love: Anxiety About ICU Visitor Restrictions

The medical team may have limited time to provide information, which may be highly technical, that the surrogate needs to make healthcare decisions for the patient. Additionally, the presence of other patients and visitors in the confined space of an ICU can limit the ability of surrogates to share their emotions and the ability of friends and family to provide emotional support. This study identified two subthemes (Figure 1).

#### 3.3.1. Persistent Smog

A patient in critical condition often requires rapid and uninterrupted medical diagnosis, treatment, and decision-making. The medical team may have very little time to provide a surrogate with the information needed to make a decision about a patient who is in critical condition. Additionally, the medical team may use rough language and behavior that can hurt the feelings of the surrogate, especially surrogates who have relatively limited medical knowledge and information. For example, Subject H mistakenly purchased a large amount of protein supplement and was distressed to find that it was not required. Subject H said, “The nurse said the patient needed two cans a day, so I bought 30 cans. The nurse then said I should have thought about it before I bought so much. She said I couldn’t get a refund. I told her not to worry and that I would not return it.”

Most people have difficulty understanding unfamiliar medical concepts. Surrogates who have low education levels and limited medical knowledge may not even know what questions to ask in the high-pressure environment of a hospital ICU. Subject B mentioned, “The doctor said that I would not understand because I do not have sufficient education. Anyway, even if I did understand, I would not know what to ask.” Misunderstandings about the treatment plan can rapidly lead to critical changes in the relationship between the surrogate and the medical team. Subject G said,

“My father-in-law underwent a full body examination every year. The first year, the image was blurry, but the doctor said it was nothing. My father-in-law is a rural laborer with no medical knowledge. After the doctor looked at the report, he did not even tell us to go to a bigger hospital for another image. He just kept saying many people have blurry images and they are fine. So, we just kept thinking this way. It was not until last year that we realized this was not right.” Surrogates often recalled how the patient felt disappointed during the diagnostic process.

#### 3.3.2. Blockaded Longing

Although all ICUs have visiting hours, the visiting hours may not be long enough to enable the patient and surrogate to form an emotional bond or to help each other understand the current condition of the patient. For one patient in this study, Subject C was the current surrogate and had been the caregiver for the patient almost 10 years earlier. The first time the patient was in critical status, endotracheal intubation and a transfer to ICU were required. Subject C said, “You guys tie him up when he is staying here. You don’t let us accompany him. You can see it’s hard for him to breathe when he gets emotional. We can help you take care of him, but you won’t let us.” When medical care can no longer maintain the health status of the patient, surrogates and the medical team must begin discussion of hospice care. Again, the medical team may not allow the surrogate to accompany the patient. When the medical team suggested transferring the patient to in-hospital hospice care, Subject G quietly asked, “Does hospice care need to be provided here? Can he be transferred elsewhere?”

### 3.4. Suffocating Love: Entanglement in Decision-Making

During the decision-making process, the surrogates and the medical team rarely interact as equals. Surrogates may lack the medical knowledge and communication skills needed to participate effectively in decision-making, especially in the emergent, fast-paced environment of the ICU. Family relationships unique to Chinese culture and emotional entanglements among family members may prevent surrogates from thinking logically when healthcare decisions must be made. This theme revealed three subthemes: chasm, unpredictability, and tangled limbs (Figure 1).

#### 3.4.1. Chasm

A chasm between the thoughts of the surrogate and the thoughts of the medical team may appear during the decision-making process. For surrogates who have limited medical knowledge, self-doubt, and hesitancy can become major obstacles in the decision-making process. Subject A was concerned that the patient could not tolerate the procedure and refused further examination of the patient. Subject A said,“He has already done this 4–5 times, and he has done TUNEL [trans catheter arterial embolization] on two consecutive days. Sometimes I just want everything to go smoothly, but he received an injection of a very strong drug. I heard this drug should only be used once in a while. So I said no. But the doctor wanted to do it. This is a situation that we do not want.”

After making decisions for the patient in the acute phase of the disease, some surrogates experienced doubt about the decision and began to question the suggestions of the medical staff. Subject F complained that the medical staff did not provide a timely response to his questions about follow-up care and treatment, “After surgery, no one explained how to maintain oral hygiene. That is what I am most worried about. The doctor did not mention whether further chemotherapy or radiotherapy is needed. Why don’t they explain what further treatment is needed?”

#### 3.4.2. Unpredictability 

For surrogates, understanding the possible results of a treatment is similar to making a guess about something that is unpredictable. That is, even in the high technology era of today, surrogate decision-making is as difficult as speculating about whether a rainbow will occur after a rain, how it will look, and how long it will last. Making a healthcare decision is particularly difficult when the surrogate knows that a poor decision made under mental distress may cause the patient pain, injury, or even death. For example, when Subject E had to decide whether the patient should undergo surgery, Subject E said, “I fear that, if he does not get well after the surgery, I will not be able to care for him because I have to work. I would not be able to live on if anything happened to him. He is stable now, which is good. Surgery, of course, is very risky. I have seen too many bad outcomes of surgery.”

#### 3.4.3. Tangled Limbs

A well-recognized feature of Chinese culture is the need to balance the concerns of the immediate family with the concerns of the extended family [26]. For surrogates who must make healthcare decisions, this tension can cause self-doubt and hesitancy. Surrogates may be tormented by this constant tension between the need to act rationally and the need to be sensitive to family members. Subject E described feelings of tension he felt when he had to decide whether the patient should undergo endotracheal intubation, “I told them not to place the tube because he would not get better. Since no one wanted to say it, I had to say it, even though I knew everyone might hate me later. I did not want my dad to suffer anymore.” The interview results show that the feelings of family members were a major consideration during decision-making by surrogates. In some cases, the surrogate deferred to other family members, even if the surrogate disagreed. Conflicts were most likely to occur when the surrogate made a decision for a patient who was in critical condition and required an invasive procedure such as surgery, endotracheal intubation, gastroscopy, or cardiopulmonary resuscitation.

## 4. Discussion

Figure 1 is the qualitative model of the decision-making process of surrogates in this study. Decision-making by a surrogate was affected by love for the patient and the close personal relationship with the patient. Four effects of this close relationship were identified: (1) Use love to resist: internal angst; (2) Allow an angel to spread love among us: memories and emotional entanglements; (3) Dilemmas of love: anxiety about ICU visitor restrictions; and (4) Suffocating love: entanglement in decision-making.

The surrogates in this study reflexively blamed themselves and hoped to reduce or compensate for the pain and suffering experienced by the patient. In some cases, the feeling of reluctance to make decisions further evolved into self-blame and then feelings of internal angst, which they described as a heartbreaking situation. Research shows that approximately 30% of surrogates experience anxiety and depression when a family member is in ICU, but the anxiety and depression taper off during the ICU stay [27]. Surrogates in this study experienced inner conflict and hesitance when they were required to make decisions that would result in physical disfigurement of the patient. The surrogates in this study also perceived that limited visiting hours weakened their emotional bonds with the ICU patients. Limited contact with patients induced mental distress in the surrogates and indirectly induced negative feelings during decision-making.

Each surrogate considered the patient an angel that the family needed to be complete. The family members described how their memories of the patient, whether happy or sad, were bits and pieces of their lives. Their positive and negative emotions coincided with positive and negative changes in the condition of the patient, and they expressed fears of being alone if the patient left them. The Confucian value system evolved from the need to maintain complex social relationships and to guide behavior and interactions among family members. That is, Confucian guidelines for behavior and conduct are intended to maintain strong family relationships and good public perceptions. Compared to westerners, however, Chinese populations tend to be more reserved in their expressions of love and other emotions. The results revealed that surrogates informally tended to avoid discussing certain highly stressful matters such as a perceived lack of family support, a poor relationship with the patient, and progression of the disease from onset to ICU admission. Ji (2012) used a phenomenological methodology and performed in-depth interviews to explore the experience of nine surrogates of ICU patients. The theme of “Being in a constantly fearful and pressured state” and its subtheme of “Family member and medical activity assistance” emerged in their study. The authors described how insufficient family support and conflicts between the family and the medical staff can induce negative feelings such as fear and psychological distress [28].

Surrogates of certain populations such as DINKs or new immigrants may feel extreme mental and physical exhaustion when no other relatives or friends are available to provide support and assistance. For many populations, especially in Asia, children are expected to provide lifelong care and support for their parents. However, this responsibility can be a heavy emotional burden. The emotional longings and desires of surrogates of cancer patients are rarely considered by the medical team, and limited ICU visiting hours deprive surrogates of their emotional bonds with the patients. The ICU team can help them by referring them for psychotherapy or other social services or by encouraging them to seek emotional support from friends and relatives.

The major sources of negative emotions for surrogates in this study were the limited visiting hours and the enclosed environment of the ICU, which deprived surrogates and patients of their emotional connection and caused a constant yearning for each other. This feeling of yearning, coupled with changes in the physical appearance of the patients related to disease or treatment, often caused the surrogates to experience tension and shock, which impaired their relationship with the medical team. Disrupted communication and connection with an ICU patient can cause family members to lose confidence in their ability to make healthcare decisions [15]. The needs and longings of surrogates are not prioritized by medical teams. For example, medical personnel who are focused on providing rapid and effective treatment for an ICU patient may not have time or energy to consider the psychological distress experienced by a surrogate who observes physical changes in the patient, such as large surgical wounds, severe bruises, or edema caused by procedures such as endotracheal intubation or repeated placement of other invasive devices. Surrogates who perceive a lack of concern about their distress may then experience feelings of resentment, which can negatively affect their decision-making capability and cause them to experience doubt and unease about the medical team. These changes can cause negative emotions such as sadness and regret as well as suspicion about the competency of the medical team [29,30].

This study revealed that conflicts between rationality and sentimentality can impair decision-making, not only when the surrogate perceives a gap between the knowledge of the medical team and the information the medical team actual conveys, but also when the surrogate experiences complex emotional entanglements with the patient. Dionne-Odom et al. (2015) explored and compared decision-making processes in surrogates for ICU patients in their last stages of life [16]. The discussed how surrogate decision-making is affected by painful affections uncertainty about the medical treatment due to a knowledge gap, e.g., a surrogate may be concerned that the patient cannot tolerate a painful medical procedure. Specifically, the author discussed how “painful affections” decrease the willingness of surrogates to authorize further treatment for the patient. For surrogates, these relationships and entanglements resemble a net with which the surrogate must disassociate in order to make a rational decision.

The interview results also revealed that a struggle for power among family members often causes conflict during the decision-making process. Family demands are a well-recognized phenomenon in the “moral and emotional concepts” factor. Surrogates must consider the demands and expectations of all family members so that the treatment decision is consensual [16]. Su (2014) conducted focus group interviews with 69 surrogates and found that communication, emotions, and conflicts are the three most important factors in the surrogate decision-making process. The struggle for power among family members with different thoughts on medical treatment for the patient is a major cause of conflict during the decision-making process [5,18] Therefore, support from family members is essential for effective surrogate decision-making. However, displays of affection and emotions such as love and gratitude are much more restrained in Chinese culture in comparison with western culture [31]. Health professionals can improve the decision-making process and help families reach a consensus by referring them for counseling. A notable cultural difference in surrogate decision-making is that surrogates in Asia tend to consider the feelings and concerns of other family members and the need to reach a consensus. In contrast, surrogates in other countries tend to focus on choosing the best treatment option for the patient. For medical teams, the main concern is rapidly and efficiently diagnosing the patient and then providing treatment in the high-stress and fast-paced environment of an ICU. Communication with surrogates is not prioritized, which increases the risk of a decision-making conflict.

The risk of conflict can be reduced by clear communication that gives surrogates the information and confidence they need to understand evolving conditions during the decision-making process. Medical teams in ICUs should share their professional opinions with surrogates and should invite surrogates to ask questions about treatment. However, information should be presented in layman terms whenever possible. Visual aids can also be helpful for discussing technical matters. For example, radiologists often use simple drawings on paper or whiteboards to explain radiograph findings [32]. The medical team should also schedule time to meet with surrogates and family members to discuss the medical plan, to address their concerns and needs, and to confirm their understanding [32]. Scheduled meetings would also assist the family members and the surrogate in making consensual decisions.

### Implications for Practice

We have four suggestions for decreasing surrogate decision-making conflict in the ICU. First, the ICU team should help surrogates by clarifying whether changes in physical appearance are short-term or long-term changes, especially after the patient undergoes an invasive procedure that substantially changes the physical appearance of the patient. Second, the ICU medical team should, as early as possible, identify surrogates who have limited support systems and refer them to social workers. Third, visiting hours for surrogates should be flexible. Flexible visiting hours would enable surrogates to maintain emotional bonds with ICU patients by providing opportunities to assist with simple tasks, e.g., sending text messages, and would enable surrogates to spend time caring for patients. Last, hospitals should provide a comfortable rest area near the ICU where the surrogate and the family can discuss medical information and treatment options.

A limitation of this study is that the sample was recruited from a single medical center, which limits the potential generalizability of the results. Factors that affect the decision-making process in healthcare surrogates may differ in other geographic regions due to differences in cultural characteristics and differences in ICU visitor policies and other hospital policies. Thus, this study should be replicated in samples of surrogates of cancer patients in ICUs in different hospital levels and in different geographic regions. All participants in this study were young or middle-aged adults, which is another limitation of this study. Additionally, the long duration of the interviews (40–60 min) may have inconvenienced or distressed the surrogates, which could have affected their interview responses. Another limitation is that, even when interviews were scheduled in advance, the emotional impact of external conditions (e.g., displays of emotional distress by other ICU visitors or a life-threatening condition observed in another ICU patient) at the time of the interview may have caused interview bias. To minimize data collection bias, future studies should perform two separate interviews for each participant. Observing the emotional impact of decision-making in healthcare surrogates, particularly in Chinese populations, is very challenging in quantitative research. Further studies are needed to perform qualitative research in a larger and more diverse population of ICU patients.

## 5. Conclusions

This study of the healthcare decision-making process for ICU patients revealed that surrogates experienced four types of love: (1) Use love to resist. The surrogates reflexively blamed themselves and hoped to reduce or compensate for the pain and suffering experienced by the patient. (2) Allow an angel to spread love among us. The surrogate and the patient accompanied each other through their memories of the decision-making process. (3) Dilemmas of love. The emotional longings and desires of surrogates of cancer patients were rarely considered by the medical team, and limited ICU visiting hours deprived surrogates of their emotional bonds with the patients. (4) Suffocating love. Conflicts between rationality and sentimentality impaired decision-making. A struggle for power among family members often caused conflict during the decision-making process. Since ICU visitor restrictions prevented the surrogate from accompanying the patient at all times, surrogates were left alone with their worries. Surrogates could not always predict the outcome of a healthcare decision. Decision-making was negatively affected by the gap between the knowledge of the medical team and the knowledge of family members and also by negative emotions generated by poor relationships among family members. This study also found that decision-making was particularly difficult for surrogates of patients in certain populations that tend to have limited social support (e.g., new immigrants, DINKs, and gay couples without relatives). Certain characteristics of Chinese culture, e.g., restrained and/or implicit expression of emotions, were another barrier to decision-making [31]. The results of this in-depth analysis of surrogate decision-making can help medical teams understand the shock and internal conflict experienced by surrogates during decision-making and provide a reference that medical staff can use to improve their communications with surrogates.

## Figures and Tables

**Table 1:** Characteristics of surrogates and patients (n = 8).

ID	Relationship to Patient	Age	Employment Status	Education Level	Duration of Interviews (min)	Number of Interviews	Cancer Type	Cancer Stage	ICU Stay (Days)	Follow-Up Site
A	Grandson	36	Full-time	University	20, 37	2	Lung	3	21	Nursing institution
B	Spouse	64	Full-time	Senior high school	43	1	Lung	3	6	Ward
C	Spouse	63	Full-time	University	57	1	Breast	1	5	Ward
D	Daughter	49	Full-time	University	65	1	Liver	4	3	Death
E	Son	34	Full-time	Senior high school	55, 41	2	Pancreatic	4	11	Death
F	Spouse	51	Full-time	Senior high school	56, 60	2	Buccal	4	5	Ward
G	Daughter- in-law	44	Housekeeper	Senior high school	60, 24	2	Lung	3	25s	Death
H	Spouse	43	Part-time	No formal education	35	1	Lung	2	23	Ward

NOTE: ID = identification, ICU = intensive care unit
